# Effect and Mechanism of *Mycobacterium tuberculosis* Lipoprotein LpqH in NLRP3 Inflammasome Activation in Mouse Ana-1 Macrophage

**DOI:** 10.1155/2021/8239135

**Published:** 2021-01-04

**Authors:** Liting Liu, Kaixin Zhai, Yue Chen, Xiaowen Chen, Guofu Wang, Lixian Wu

**Affiliations:** Department of Medical Microbiology and Immunology, Clinical Molecular Immunology Innovation Team, Dali University, Dali 671000, China

## Abstract

The study is aimed at investigating the role and mechanism of LpqH of *Mycobacterium tuberculosis* in the activation of NLRP3 inflammasome in mouse Ana-1 macrophages. ExPASy-ProtParam, PHYRE2, ABCpred, and SYFPEITHI were used to predict and analyze the physicochemical properties, protein structure, and B cell/T cell-associated epitopes of LpqH protein. The recombinant LpqH protein was purified, and its immunoreactivity was analyzed with western blot. The LPS-treated mouse Ana-1 macrophages were incubated with purified LpqH protein directly. The expression of NLRP3, ASC, and caspase-1 protein was detected by western blot. The secretion of IL-1*β* was detected by ELISA, and LDH was detected by a kit. Cell death was detected by flow cytometry. LpqH consisted of 159 amino acids and was a hydrophobic protein with stable properties. Its secondary structure contained 47% random coils, 53% *β*-sheets, and 3% *α*-helix. The tertiary structure showed a relatively loose spatial conformation. Additionally, it had 8 B cell epitopes (score > 0.8) and 10 CTL cell epitopes (score ≥ 20). The recombinant LpqH, which had strong immunoreactivity, significantly increased the levels of NLRP3, ASC, and caspase-1 p20 (*P* < 0.01) and promoted the secretion of IL-1*β* by the cells (*P* < 0.01). In addition, high concentration of KCl significantly inhibited the effect of LpqH on mouse Ana-1 macrophages and reduced the expression of NLRP3, ASC, and caspase-1 p20 (*P* < 0.01). However, there was no significant change in LDH (*P* > 0.05). Meanwhile, LpqH protein did not cause additional cell death (*P* > 0.05). LpqH protein has good immunogenicity and can activate the NLRP3 inflammasome through the potassium efflux pathway without causing cell death.

## 1. Introduction

Tuberculosis (TB), caused by *Mycobacterium tuberculosis* (MTB), is an infectious disease that seriously harms human health. TB is widely distributed throughout the world and is one of the top ten causes of death worldwide. According to the WHO statistics, one-third of the world's people have been infected with MTB, and 90% have latent MTB infection [1]. According to a 2018 World Health Organization (WHO) report, 10 million people had tuberculosis and 1.6 million died of the disease in 2017 [1]. TB is particularly serious in developing countries, and China is still one of the countries with a high burden of TB. The widespread infection of TB is directly related to poor treatment, drug resistance, and the limitations of vaccines [1]. Therefore, there is an urgent need to develop new therapeutic drugs and more effective protective vaccines.

Inflammasome is a macromolecular complex assembled by multiple proteins and is activated when cells are infected or stimulated [2]. Inflammasome is activated by different recognition receptors, which determines its different structures and functions, such as NLRP1, NLRP3, AIM2, and NLRC4 [2]. NOD-like receptor-family pyrin domain-containing 3 (NLRP3) inflammasome is formed by the aggregation of NLRP3 protein, ASC protein, and procaspase-1 protein [3]. Procaspase-1 is activated during inflammasome assembly and then cleaved into active caspase-1 p20 [3]. After activation, caspase-1 cleaves pro-IL-1*β* and pro-IL-18 to form mature IL-1*β* and IL-18, which in turn causes infiltration of inflammatory cells and a more extensive inflammatory response [3]. Two signals are required for the release of proinflammatory cytokines (such as IL-1*β* and IL-18) by activation of the NLRP3 inflammasome. The first signal is the “start signal.” Activation of the Toll-like receptor or IL-1 receptor upregulates the expression of NLRP3 protein, pro-IL-1*β*, and pro-IL-18. The second is the “activation signal.” Caspase-1 activated by PAMPs or DAMPs cleaves pro-IL-1*β* and pro-IL-18 to form mature IL-1 and IL-18 [4]. Recent studies have found that the NLRP3 inflammasome is related to MTB infection [5–7]. Studies have shown that attenuated MTB H37Ra strain, virulent H37Rv strain, nontuberculous mycobacteria (such as *Mycobacterium abscessus* and *Mycobacterium kansasii*), and ESAT-6 protein of MTB can activate NLRP3 in macrophages, which in turn causes the inflammatory response [6–11].

LpqH protein is a cell wall lipoprotein of MTB, also known as 19 kDa protein, and is an agonist of the host Toll-like receptor 2 [12]. It plays an important role in mediating the interaction between the host and the pathogen and promotes the release of proinflammatory cytokines by the host cells [13], which in turn induces programmed cell death processes such as apoptosis and autophagy and enhances the host cells' ability to clear the pathogens [14–16].

In this study, bioinformatics was performed to analyze the physicochemical properties of the LpqH protein. Then, the LpqH protein was expressed and purified through a prokaryotic expression system. The role and mechanism of LpqH on LPS-treated mouse Ana-1 macrophages were further investigated and discussed. These results may lay a theoretical foundation for the development of prevention and treatment methods of TB via targeting LpqH.

## 2. Materials and Methods

### 2.1. Bioinformatics Analysis

ExPASy-ProtParam (https://web.expasy.org/protparam/), PHYRE2 (http://www.sbg.bio.ic.ac.uk/phyre2/html/), ABCpred (http://crdd.osdd.net/raghava/abcpred/), and SYFPEITHI (http://www.syfpeithi.de/) were used to analyze the physicochemical properties, protein structure, and B/T cell epitope of LpqH protein.

### 2.2. Expression and Purification of LpqH

The recombinant plasmid pET-28a-LpqH constructed by Shanghai Sangon Biotech (China) was transformed into BL21 *E. coli*. After induction, *E. coli* were collected and sonicated. Thereafter, the proteins were further purified by Ni-NTA affinity chromatography with gradient urea buffer (Kangwei Century Biotechnology Co., Ltd., Beijing, China). After removal of endotoxin by the Endotoxin-Be-Gone kit (BBI Life Sciences Co., Ltd., Shanghai, China), the protein concentration was measured using a BCA protein concentration determination kit (Solibao Science & Technology Co., Ltd., Beijing, China). Finally, the purified protein was filtered through a 0.22 *μ*m filter and stored at −80°C for later use. The antiserum obtained from immunized rabbits was used as the primary antibody, and the purified LpqH protein was verified by western blot.

### 2.3. LpqH Treatment of Ana-1 Macrophages

The mouse macrophage cell line Ana-1 was cultured in RPMI-1640 medium (Gibco) with 10% fetal bovine serum (FBS, PAN Biotech, Aidenbach, Germany) and 1% penicillin-streptomycin (Beijing Solarbio Science & Technology Co., Ltd.). Cells were first primed with lipopolysaccharide (LPS, 1 *μ*g/mL, Beijing Solarbio Science & Technology Co., Ltd.) for 4 h and then divided into the negative control group, the LpqH treatment group, and the KCl treatment group. Cells in the negative control group were treated with BSA for 2 h. The LpqH treatment group was treated with different concentrations of LpqH (0.1 *μ*g/mL, 0.5 *μ*g/mL, and 1.0 *μ*g/mL) for 2 h. The KCl treatment group was treated with 50 mM KCl (Beijing Solarbio Science & Technology Co., Ltd.) + 1.0 *μ*g/mL LpqH protein for 2 h. In addition, ATP was added as a control group for lactate dehydrogenase (LDH) measurement and flow cytometry.

### 2.4. Western Blot

Total proteins were extracted from Ana-1 macrophages. The extracted proteins were subjected to SDS-PAGE electrophoresis and transferred to membranes. After blocking, the membrane was incubated with primary antibodies against NOD-like receptor-family pyrin domain-containing 3 (NLRP3) (CST, Danvers, MA, USA), ASC (CST, Danvers, MA, USA), caspase-1 p20 (Proteintech Group Inc., Wuhan, China), and *β*-actin (CST) at 4°C overnight and then incubated with secondary antibody (1 : 5000; GenScript Biotechnology, Nanjing, China) at room temperature for 2 h. After washing, chemiluminescence reagent was added for color development. *β*-Actin was used as the internal control. The gray value of the band was analyzed by ImageJ software (ImageJ v1.8.0, US National Institutes of Health). Relative protein level was expressed as the ratio of the expression of target protein (NLRP3, ASC, and capsase-1 p20) to that of *β*-actin.

### 2.5. LDH Measurement

LDH was detected by LDH detection kits (Beijing Solarbio Science & Technology Co., Ltd., Beijing, China) according to the instructions.

### 2.6. ELISA

The cell supernatants of each group were collected separately, and IL-1*β* in the cell supernatants was detected according to the instructions of the mouse IL-1*β* ELISA kit (Elabscience Biotechnology Co., Ltd., Wuhan, China). Briefly, after the sample was incubated at 37°C for 90 min, 100 *μ*L of biotinylated antibody was added. After incubating at 37°C for 60 min, 100 *μ*L avidin-conjugated HRP was added and incubated at 37°C for 60 min. After washing 5 times, 90 *μ*L of TMB substrate solution was added and the reaction was terminated after incubating at 37°C for 15 min. The OD value was measured with a microplate reader at a wavelength of 450 nm, and the concentration was calculated.

### 2.7. FACS

Cells were collected, centrifuged, and resuspended in 1 mL cell staining buffer (Beijing Solarbio Science & Technology Co., Ltd., Beijing, China). After incubating with 5 *μ*L Hoechst and PI (Beijing Solarbio Science & Technology Co., Ltd., Beijing, China) at 4°C for 30 min, the cells were detected by flow cytometry.

### 2.8. Statistical Analysis

All data were analyzed by SPSS 22.0. Data were expressed as mean ± SD. Two-tailed one-way analysis of variance (ANOVA) with multiple comparison post hoc analysis was used. A *P* value < 0.05 was considered statistically significant.

## 3. Results

### 3.1. Bioinformatics Analysis of LpqH Protein

**Figure 1:**
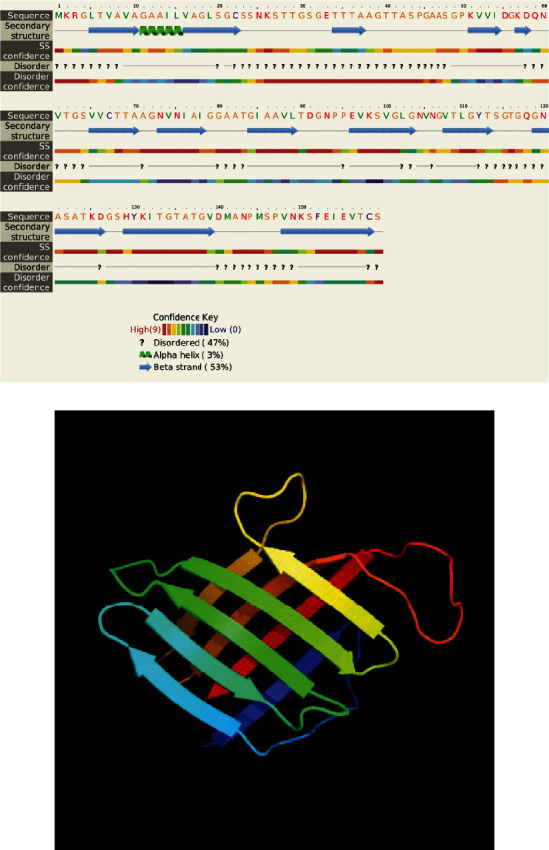
Prediction of LpqH protein structure. (a) Schematic diagram of the secondary structure. The secondary structure mainly includes the secondary structure of some proteins such as random coil, *β*-sheet, and *α*-helix. (b) Prediction of the tertiary structure. The overall structure of the protein shows that the protein structure is loose, which is conducive to the formation of more epitopes.

Analysis of physical and chemical properties showed that the half-life of LpqH was 30 h. The coefficient of instability and fatness was 24.5 and 76.10, respectively, indicating that the LpqH protein is relatively stable. Protein secondary structure showed that LpqH contained 47% random coil, 3% *α*-helix, and 53% *β*-sheet (Figure 1(a)). The tertiary structure model (Figure 1(b)) showed that the LpqH protein structure was relatively loose, which helps to form epitopes, thereby promoting the antigen-antibody binding and the generation of immune response. Cell antigen epitope prediction results showed that the protein contained many antigen epitopes (Tables 1 and 2), including 8 B cell epitopes (score > 0.8) and 10 CTL cell epitopes (score ≥ 20). These results suggest that LpqH protein has good antigenicity and has the potential to become a new target for TB vaccine research.

### 3.2. Expression, Purification, and Identification of LpqH Recombinant Proteins

**Figure 2:**
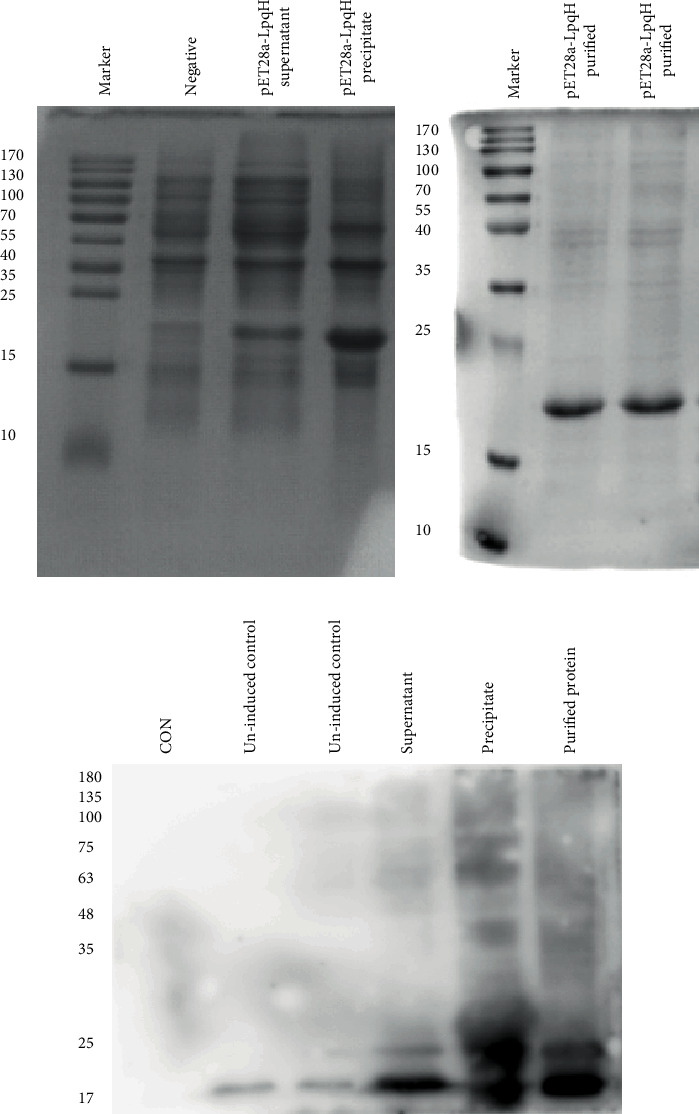
Expression, purification, and identification of LpqH. (a) The recombinant LpqH protein was expressed in *E. coli* BL21 (DE3). Analysis of expression of fusion protein LpqH by SDS-PAGE. (b) Analysis of purified fusion protein LpqH by SDS-PAGE. M: marker. (c) Identification of target protein LpqH by western blotting.

The LpqH protein expression was induced for 4 h at 37°C and with 1.0 mM IPTG. The recombinant LpqH protein was expressed as a His-tagged protein, and the His-tag was not removed. Compared with the negative control (uninduced bacteria), the induced bacteria showed a distinct specific protein band at a position of about 20 kDa (Figure 2(a)). The expressed protein was mainly inclusion bodies. The purified protein was obtained after urea denaturation lysis and renaturation purification on a Ni-NTA affinity chromatography (Figure 2(b)). The LpqH protein was identified by using a polyclonal antibody obtained by immunizing rabbits with H37Rv strain of MTB. The result showed that a specific band appeared around 20 kDa (Figure 2(c)). The above results show that the LpqH protein is successfully expressed and purified and has good immunogenicity.

### 3.3. Effect of LpqH on NLRP3, ASC, and Caspase-1 p20 in Ana-1 Macrophages

**Figure 3:**
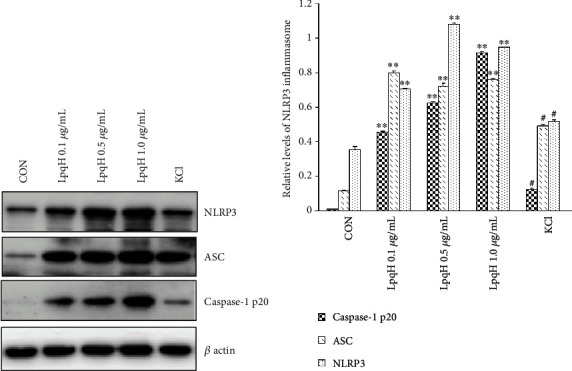
Effect of LpqH on the expression of NLRP3, ASC, and caspase-1 p20 in Ana-1 macrophages. Cells were first primed by LPS and then were grouped into different groups, including the negative control group, or treated with LpqH (0.1 *μ*g/mL, 0.5 *μ*g/mL, and 1.0 *μ*g/mL) for 2 h, or treated with LpqH 1.0 *μ*g/mL and 50 mM KCl for 2 h. Western blot was used to detect the expression of NLRP3, ASC, and caspase-1 p20. ^∗∗^*P* < 0.01 compared with control; ^#^*P* < 0.01 compared with LpqH 1.0 *μ*g/mL.

Western blot was performed to detect NLRP3, ASC, and caspase-1 p20 protein expression. As shown in Figure 3, compared with the negative control group, the expression of NLRP3, ASC, and caspase-1 p20 in the LpqH-treated group was significantly increased (*P* < 0.01) in a dose-dependent manner. However, the levels of NLRP3, ASC, and caspase-1 p20 were significantly reduced after KCl treatment (*P* < 0.01).

### 3.4. Effect of LpqH on IL-1*β* and LDH Secretion by Ana-1 Macrophages

**Figure 4:**
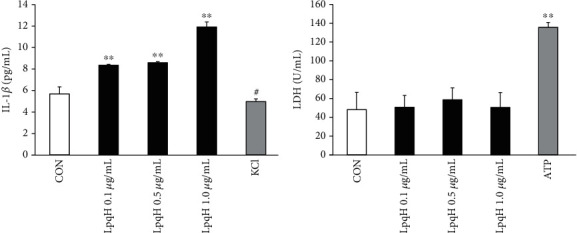
Effect of LpqH on the secretion of IL-1*β* and LDH by Ana-1 macrophages. Cells were first primed by LPS and then were grouped into different groups, including the negative control group, or treated with LpqH (0.1 *μ*g/mL, 0.5 *μ*g/mL, and 1.0 *μ*g/mL) for 2 h, and treated with LpqH 1.0 *μ*g/mL and 50 mM KCl or 5 *μ*M ATP for 2 h. ELISA was performed to detect levels of (a) IL-1*β* and (b) LDH in the culture supernatant. ^∗∗^*P* < 0.01 compared with control; ^#^*P* < 0.01 compared with LpqH 1.0 *μ*g/mL.

To determine the effect of LpqH on IL-1*β* and LDH, ELISA was conducted. As shown in Figure 4(a), compared with the negative control group, LpqH significantly promoted IL-1*β* secretion by macrophages. After treatment with KCl, the ability of LpqH to promote the secretion of IL-1*β* by macrophages was significantly reduced (*P* < 0.01). However, there was no significant difference in the LDH level among different groups (*P* > 0.05) (Figure 4(b)). These results indicate that LpqH caused increased IL-1*β* secretion.

### 3.5. Effect of LpqH on Ana-1 Macrophage Death

**Figure 5:**
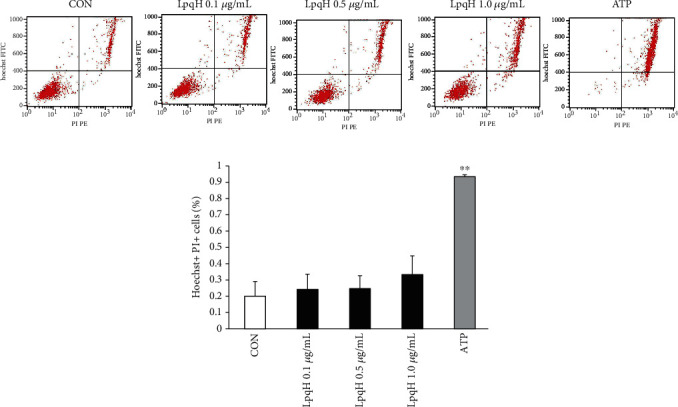
Effect of LpqH on necrosis of Ana-1 macrophages by flow cytometry. Cells were not treated (negative control group), or treated with LpqH (0.1 *μ*g/mL, 0.5 *μ*g/mL, and 1.0 *μ*g/mL) for 2 h, or treated with 5 *μ*M ATP for 2 h. Flow cytometry was used to detect cell necrosis. (a) Representative flow cytometry results. (b) Quantitative flow cytometry results. ^∗∗^*P* < 0.01 compared with other groups.

To detect the effect of LpqH on necrosis of Ana-1 macrophages, flow cytometry was performed. As shown in Figures 5(a) and 5(b), compared with the negative control, LpqH did not significantly affect macrophage death (*P* > 0.05). Therefore, LpqH has no significant effect on cell death.

## 4. Discussion

Studies have found that inflammasome played an important role in the host immunity against MTB infection, and mice deficient in IL-18, IL-1*β*, or IL-1 receptor type I (IL-1R1) are more susceptible to MTB [17, 18]. Meanwhile, NLRP3- or ASC-deficient animals have increased susceptibility to TB due to impaired inflammasome formation [19, 20]. However, till now, we know little about the specific mechanism of inflammasome activation during MTB infection. In this study, we found that LpqH relied on potassium efflux to activate NLRP3 inflammasome.

LpqH is a lipoprotein in the cell wall of MTB. It is originally identified in crude bacterial extracts recognized by mouse antibodies and considered to be one of the main antigens of MTB [21]. LpqH is expressed by MTB and other slow-growing pathogenic mycobacteria such as *Mycobacterium bovis* BCG and *Mycobacterium avium-intracellulare*, but not by fast-growing nonpathogenic Mycobacteria such as *Mycobacterium smegmatis* or *Bovine Mycobacteria* [22]. Research on the LpqH protein has shown that it plays a complex role in the interaction between bacteria and the host immune system [13–16, 23, 24], but its relationship with inflammasome is currently not reported in the literature. Through bioinformatics analysis, we found that the LpqH protein had good antigenicity, which will be one of the key proteins in understanding the relationship between MTB infection and the host.

NLRP3 and AIM2 inflammasome have been found to play a vital role in MTB-induced immunity. Among them, NLRP3 inflammasome is one of the mostly studied and is activated by a wide range of substances [5]. Here, we expressed and purified LpqH to act on mouse macrophages *in vitro* and detected an increase in the expression of the main components of NLRP3. Moreover, LpqH promoted caspase-1 p20 in a dose-dependent manner and also enhanced IL-1*β*. At present, the research on the activation mechanism of NLRP3 inflammatory bodies is still unclear, and there are many hypotheses. Among them, there is a view that NLRP3 triggers an inflammatory response through the outflow of potassium ions in cells or the reduction of potassium concentration in cells [25]. After inhibiting potassium ion outflow from cells with high concentration of KCl, we found that the expression of the main component of the NLRP3 inflammasome was significantly inhibited, accompanied by decreased secretion of IL-1*β*. Therefore, we believe that the activation of inflammasome by LpqH depends on potassium efflux.

However, due to the limitations of the experimental design, there are still many shortcomings in this study, including the narrow range of LpqH protein concentration used in the experiment and the unexplained mechanism on how LpqH causes potassium ion outflow. These problems need to be further explored in the future research.

## 5. Conclusions

In conclusion, we found that LpqH activated mouse Ana-1 macrophage NLRP3 inflammasome possibly through the potassium outflow and then induced inflammatory response. Our results may help us understand the relationship between host macrophages and MTB and may provide a theoretical basis for developing new treatments for TB.

## Figures and Tables

**Table 1:** Prediction of B cell epitope of LpqH protein.

	The position of amino acids	Epitope sequences	Score
1	129	HYKITGTATGVDMANP	0.95
2	29	TGSGETTTAAGTTASP	0.87
3	110	LGYTSGTGQGNASATK	0.86
4	44	PGAASGPKVVIDGKDQ	0.84
5	81	AATGIAAVLTDGNPPE	0.83
6	51	KVVIDGKDQNVTGSVV	0.83
7	20	SGCSSNKSTTGSGETT	0.82
8	135	TATGVDMANPMSPVNK	0.82

**Table 2:** Prediction of T cell epitope of LpqH protein.

	The position of amino acids	The prediction of CTL epitope sequences of antigen	Score
1	80	GAATGIAAV	26
2	11	GAAILVAGL	25
3	53	VIDGKDQNV	22
4	4	GLTVAVAGA	21
5	77	AIGGAATGI	21
6	89	LTDGNPPEV	21
7	100	VGLGNVNGV	21
8	131	KITGTATGV	21
9	8	AVAGAAILV	20
10	140	DMANPMSPV	20

Note: cytotoxic T lymphocyte.

## Data Availability

The data that support the findings of this study are available on request from the corresponding author.
